# Mental, physical, and phychophysiological responses to FFP2/N95 face mask during HIIT in active women

**DOI:** 10.1371/journal.pone.0292061

**Published:** 2023-09-29

**Authors:** Kyran Tannion, Ricardo De la Vega, Javier Horcajo, Víctor Cuadrado-Peñafiel

**Affiliations:** 1 Department of Physical Education, Sport & Human Movement, Universidad Autónoma de Madrid, Madrid, Spain; 2 Departamento de Psicología Social y Metodología, Universidad Autónoma de Madrid, Madrid, Spain; Instituto Politécnico de Santarém: Instituto Politecnico de Santarem, PORTUGAL

## Abstract

Training systems based on high-intensity interval training (HIIT) have experienced great influence in recent years within the context of exercise and sport. This study aims to provide insight on whether the immediate outcomes (e.g., central and peripheral acute responses) may be intensified or attenuated when a HIIT protocol is performed using a FFP2/N95 face mask in active healthy adult women. In other words, it strives to provide new findings relative to the use of face masks as a potential performance enhancing tool. In the current study, the same training session was carried out on two occasions under different conditions (i.e., with FFP2/N95 and without FFP2/N95) in a cross-over experimental design. The following study variables were assessed before and after the HIIT in both sessions: Lactate, cortisol, alpha-amylase, selective attention, countermovement jump (CMJ), and power output. Additionally, central and peripheral Rates of Perceived Exertion (RPE) were assessed before and during the HIIT. This study makes novel contributions to prior research, showing that the use of FFP2/N95 face mask (vs. no mask) yielded higher alpha-amylase, selective attention, and peripheral RPE scores. No significant differences were found for lactate, cortisol, CMJ, and power output. Interestingly, central RPE scores were significantly lower under FFP2/N95 face mask (vs. no mask) condition. The main suggestion in light of these results is that researchers and practitioners should consider potential peripheral and central responses to training stimuli when using FFP2/N95 face masks.

## Introduction

Facial masks, a key preventive measure during COVID-19, have quickly become a standard practice in various settings [1]. Prior to that, studies already explored the effects of mask use during exercise and high-demand situations like military tasks [2]. This leads to the question: Can face masks influence mental, physical, and phychophysiological responses to High-Intensity Interval Training (HIIT)? HIIT involves alternating high-intensity activity with active recovery or rest and offers both physical and mental health benefits [3, 4]. While HIIT is recommended for the general population and athletes, it’s also effective for clinical groups [e.g., 5]. However, more research is needed on its psychological effects, as high intensities might induce negative responses [6].

The impact of mask use on training responses and performance enhancement remains unclear. Prior findings should be approached cautiously due to inconsistencies in the literature. Physical activity and exercise are defined variably, as well as study samples also differ. Additionally, mask types vary, including surgical, FFP2/N95, and cloth masks. Thus, research should standardize activity types, mask types, participant characteristics, and study effects at specific exercise intensities and levels, such as psychophysiological or mental [7–9]. That said, for example, some prior studies have explored the voluntary use of masks for flow restriction to enhance aerobic and anaerobic performance [10, 11].

In light of the above, more research is needed on the mental responses to HIIT performed with face masks. For instance, Rates of Perceived Exertion (RPE) is a broadly used measure. The anatomy and structure of such perception is habitually founded on sensations related to cardiopulmonary and peripheral factors (e.g., aching, strain…). In addition, RPE has mainly been utilized under two paradigms: Assessment (i.e., quantifying sensed effort due to a specific prescribed exercise) and production (i.e., prescribing exercise based on the achievement of a specific sensed effort) in contexts that imply demanding tasks. Thus, it has been classified as an indicator of perceived somatic (i.e., body-related) stress response [12]. In the present research, we propose that both mental (i.e., central) and physical (i.e., peripheral) factors should be taken into account to avoid reporting a vague explanation of fatigue. We suggest that RPE measures should involve physical and mental variables related to heterostaticness (i.e., homeostatic disturbance).

It’s crucial to grasp how the stress response system works [13]. Therefore, alongside subjective markers of homeostatic disturbance, objective indicators like cortisol levels, selective attention, and motor performance are relevant. Past studies indicate a connection between mood, cortisol, and information processing [14]. While some research suggests that stress improves selective attention by focusing on relevant tasks [15], others argue stressors hinder it due to distractions [16].

This study focuses on active healthy adult women, addressing the current gender data gap in sports science [17, 18]. Our goal is to enhance understanding of face mask use’s impact on training load in this group. This research poses the following questions for active healthy women during HIIT. Does wearing an FFP2/N95 mask (vs. No mask) lead to different:

1. metabolic (i.e., lactate) and endocrine responses (i.e., cortisol and alpha-amylase)?
2. immediate physical performance (i.e., countermovement jump and power output)?
3. selective attention as measured by the Stroop Test?
4. perceived physical (i.e., peripheral RPE) and mental fatigue (i.e., central RPE)?

Thus, our goal is to offer insights for athletes and trainers to optimize performance, adapt training, and address challenges of face mask use during intense workouts. By examining a broad range of responses, we hope to bridge a significant gap in the literature and provide a comprehensive view of the implications of face mask use during HIIT.

## Method

This study was developed according to the Standards for Ethics in Sport and Exercise Science Research [19] and in compliance with the Helsinki Declaration. It was conducted with the approval of the Research Ethics Committee of the Autonomous University of Madrid (Registration No. CEI-106-2060).

### Participants

An *a priori* analysis to estimate sample size was carried out with the G*Power software 3.1.9.7 [20]. Large effect sizes (Cohen’s f = 0.5) were anticipated. Hence, with α = 0.05, 1-β = 0.95 and r = 0.5, the minimum sample size required reported by the system was n = 16. Finally, 17 healthy volunteers participated in the project. The mean age was 51.7 ± 14.7. All were women and regular users of public sports facilities in the Autonomous Community of Madrid (Spain), as well as indoor cycling users with at least a year of experience. An essential requirement was the optimal health of the participants in the two months prior to engaging in this study. Therefore, any person with a health (physical and/or mental) problem in that space of time was considered ineligible to participate. In accordance with the guidelines outlinedfor classifying groups of female subjects in sports science, all participants met the criteria for performance level 2 (i.e., active) [21].

### Experimental design

This study was developed in a within-subject experimental design (i.e., cross-over study). Therefore, the same training session was carried out on two occasions under different experimental conditions (i.e., FFP2/N95 vs. No mask). The HIIT procedure that participants engaged in was prescribed by Physical Activity and Sports Sciences professional. It lasted a total of 25 minutes and was directed by an indoor cycling trainer. Simultaneously, the progress of the HIIT was projected live on a screen. It consisted of a brief activation (zone 3, self-regulated cycling cadence, 1 minute) and 8 high intensity peaks (zone 5, 70 rpm cycling cadence, 1 minute and 30 seconds) interspersed with 8 moderate intensity active recoveries (zone 3, self-regulated cycling cadence, 1 minute and 30 seconds). Participants were instructed, after equipping them with a Polar m-430 heart rate monitor, to maintain HR between 90–100% of their maximum HR when they were in zone 5 (i.e., high intensity bout) and between 70–80% of their maximum HR when in zone 3 (i.e., active recovery). Such a prescription was based on the evidence provided by Canário-Lemos et al. [22] regarding effective intensity control in indoor cycling. Maximum HR was estimated for each participant with the equation developed by Tanaka et al. [23]. According to the authors, such a regression line does not differ between men and women. Also, it is not influenced by large variations in terms of habitual physical activity levels. Therefore, the HIIT was adapted to each volunteer’s capability. In other words, the training load was individualized.

### Study variables and instrumentation

#### Lactate

Blood lactate concentration is a broadly measured parameter to assess metabolic demands of exercise (i.e., controlling training) and/or to predict performance [24]. In this study, it was measured with the Portable Pro Lactate Analyzer (KDK) which is precise, reliable, and shows a high degree of consistency with other lactate analyzers [25, 26]. Sample collection was performed by introducing a lactate strip into the calculator-sized instrument and then touching the strip to a drop of blood from the index finger. It is worth mentioning that measurements were done in the third minute following the last high-intensity bout of the proposed training session. Such a decision was based on the guidelines provided by Goodwin et al. [27].

#### Cortisol

The adrenal glands produce this steroid hormone to promote the body’s response to stress (organic or perceived), regulate the level of sugar in the blood and/or fight infections. Salivary cortisol responses are widely used in sports as a psychophysiological marker of stress induced situations [28]. Following the indications from prior research [e.g., 29], a saliva sample was collected. Participants were asked to passively drool into a plastic tube [30] and subsequently, its analysis was carried out in the laboratory.

#### Alpha-amylase

It has proven to be a sensitive biomarker for stress that indicates increased sympathetic nervous activity, resulting in a widely used measure in the sport and physical activity context [e.g., 31]. Its assessment was salivary, just as cortisol.

#### Countermovement jump (CMJ)

Assessed to quantify applied force capacity of the lower limbs. It was measured using the MyJump2 app which has proven to be a valid, reliable, and useful tool for measuring the vertical jump [32]. Prior to the study, two previous familiarization sessions were conducted, during which the same researcher provided feedback on the range and velocity of descent, as well as the placement of the legs during the flight and landing phases. For its assessment, participants were required to execute 5 jumps with a 30 second rest in between. The average height was computed from these 5 jumps.

#### Power output in the CMJ

Defined as the ability to produce force in a short space of time, it is a mechanical parameter highly related to sports performance [33]. It was calculated with the developed and validated equation from Samozino et al. [34].

#### Selective attention (i.e., Stroop performance)

Understood as the orientation and voluntary use of cognitive resources or of the senses towards a given stimulus (regardless of the nature). It has been proven that intrinsic fluctuations in brain activity wield influence neural mechanisms on which selective voluntary attention and cognitive control rely [35]. Such evidence is of the utmost importance when presented concurrently with what Ai et al. [36] stated based on their systematic review: HIIT tends to have a positive effect on executive functions. Selective attention was evaluated using the digitized version of the Stroop Color and Word Test (SCWT) [37] on a computer. Participants were provided with instructions to carry out the test and a 30 second trial was performed prior to each assessment to ensure its comprehension. Later, participants proceeded to perform the task. The scoring in the test was based on the guidelines set by Scarpina & Tagini [38]: correct answers in a fixed time (i.e., 1 minute) were recorded.

#### Rates of Perceived Exertion (RPE)

It is a psychophysiological marker that quantifies intensity and homeostatic disturbance (i.e., fatigue) during or due to exercise according to the judgment of the person involved in such exercise. Perceived exertion was assessed with Borg’s 6−20 scale [39]. This study distinguished between peripheral (i.e., physical effort) and central (i.e., mental effort) RPE, asking every participant about their perceived effort (i.e., either physical or mental) in each of the six measurements that were carried out. The RPE measure is a 15-point scale, beginning at 6 (i.e., *no exertion at all*) and that goes up to 20 (i.e., *maximal exertion*). Before testing, subjects were instructed on the use of the RPE scale [e.g., 40].

### Procedure

**Fig 1:**
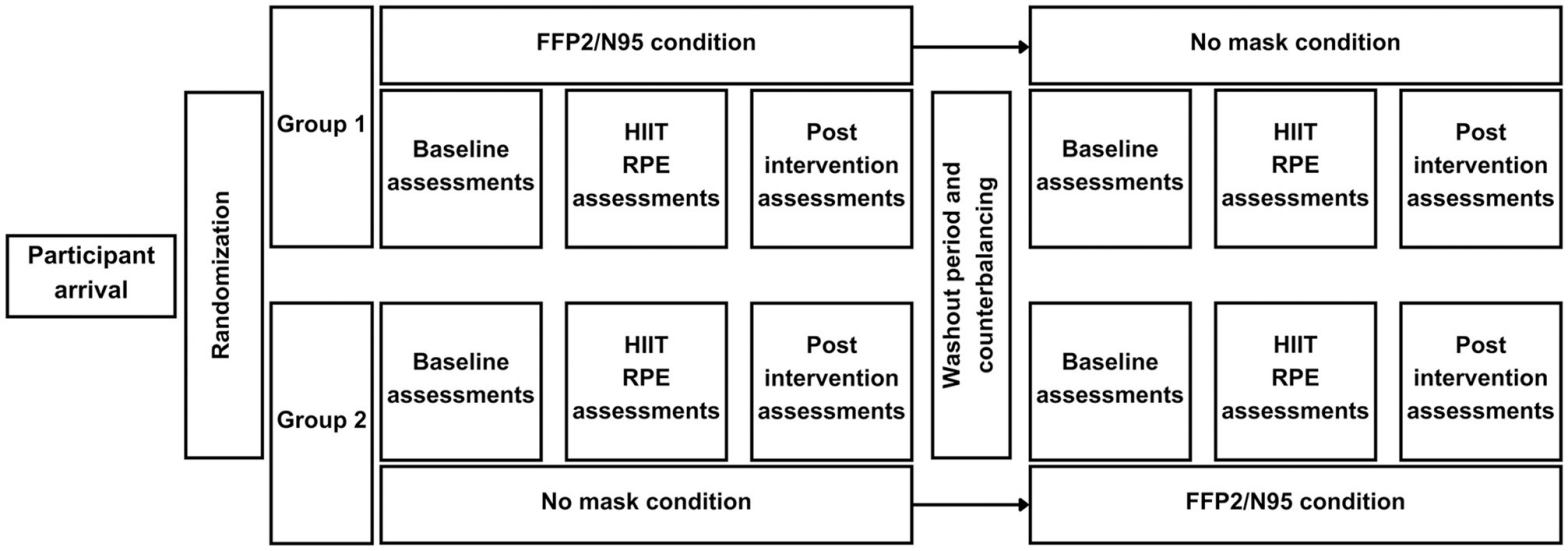
Research diagram.

The objectives, method, and procedure of this study were explained to each participant, as well as the potential risks and benefits. Prior to starting, volunteers signed an informed consent and were randomly assigned to a different condition presentation order. In addition, the conditions (i.e., FFP2/N95 vs. No mask) under which the HIIT was performed were randomized to each of these groups in the first session and counterbalanced in the second session. Such a course of action was performed according to guidelines on randomized cross-over designs [41, 42]. Study variables were measured before and after the HIIT in both sessions. More precisely, lactate, cortisol, alpha-amylase, and Stroop performance were assessed upon arrival of the volunteers. Subsequently, a brief warm-up was done (15 minutes). Such warm-up was structured and directed by a Physical Activity and Sports Sciences professional based on the work developed by Wei et al. [43]. Then CMJ, power output, and baseline central and peripheral RPE were measured. When the baseline assessment was finished, participants performed the HIIT. Central and peripheral RPE were also assessed during the HIIT. Specifically, just after the first, the third, the fifth, and the eighth high-intensity bouts. Post-intervention (i.e., HIIT) measurements of lactate, cortisol, alpha-amylase, Stroop performance, CMJ and power output were done in the same order as the baseline measurements. There was a period of 15 days between each condition (i.e., washout period). The difference between conditions relied only on wearing (vs. not wearing) a mask during the HIIT. Despite that, both sessions were carried out identically (i.e., 20ºC temperature, same day of the week, same hour of the day, same sports facility, and procedure). The research diagram summarizes the previously explained (Fig 1).

### Statistical analysis

Except for RPE, there were two measurements per condition of each study variable: one before and one after experimental intervention (i.e., training session). For the statistical analysis, an index was created for each of the study variables. That is, the index was defined as the subtraction of the baseline measurement to post experimental intervention measurement. Such an index was used as a dependent variable. Dependent variables were submitted to a 2 × 2 mixed ANOVA with Mask Use (FFP2/N95 vs. No mask) as within-participants factor and Condition Presentation Order (first session FFP2/N95 vs. first session no mask) as the between-participants factor. The statistical significance level was set at p ≤ 0.05 (α = 0.05). Effect size estimate (i.e., partial eta squared) was calculated following guidelines offered by previous research [44, 45].

## Results

**Fig 2:**
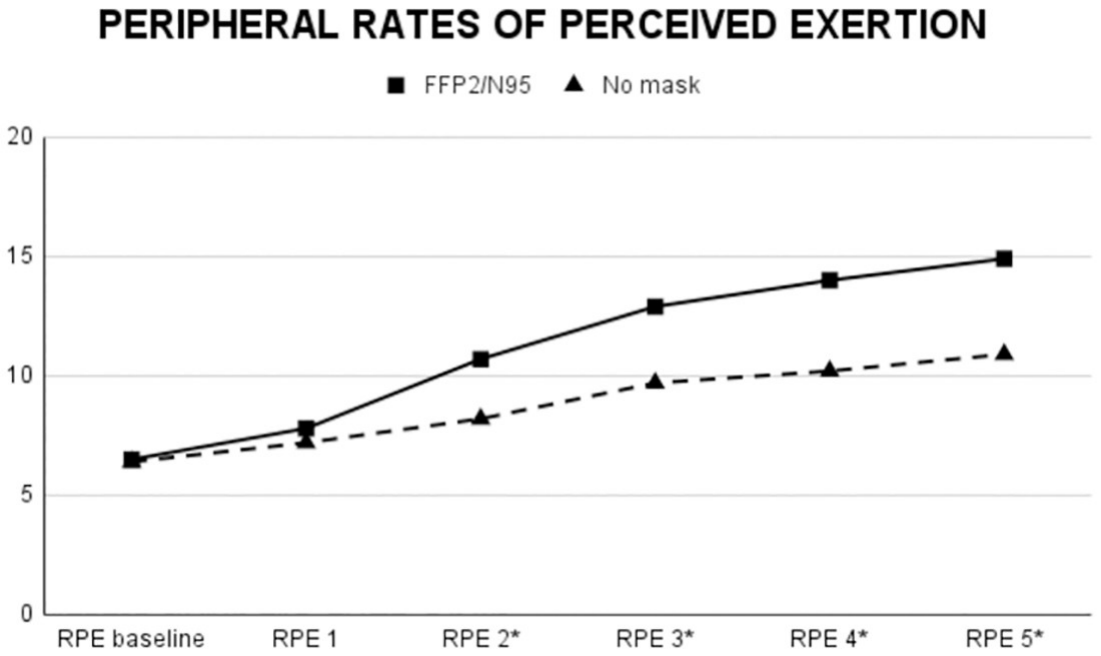
Temporal evolution of peripheral RPE values during the training session.

**Fig 3:**
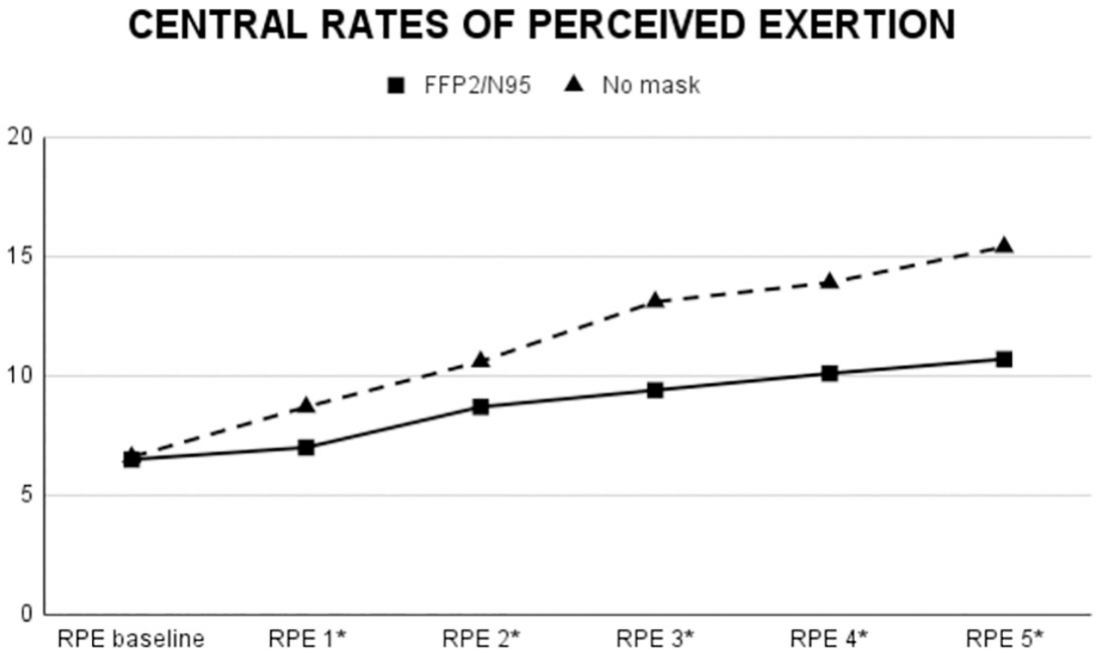
Temporal evolution of central RPE values during the training session.

Descriptive data is presented in Table 1 and the inferential statistics data is reported in Table 2. A main effect of Mask Use was found for Stroop performance and alpha-amylase. FFP2/N95 condition (vs. No mask) produced significantly (p<0.05) higher values in such dependent variables. Also, a main effect of Mask Use was detected for peripheral and central RPE. That is, among the baseline and successive five measurements, FFP2/N95 condition (vs. No mask) induced significantly (p<0.05) higher values from the second measurement after the baseline onwards in peripheral RPE (Fig 2, Table 2). This pattern was reversed for central RPE. FFP2/N95 condition induced significantly (p<0.05) lower values from the first measurement after baseline onwards (Fig 3, Table 2). No main effect of Condition Presentation Order was found for any of the dependent variables except for lactate. Mask Use x Condition Presentation Order interaction was not found for any of the dependent variables.

**Table 1:** Descriptive data (i.e., mean and standard deviation).

	FFP2/N95			No mask		
Study Variable	Baseline	Post HIIT	Index	Baseline	Post HIIT	Index
Lactate	2.5±2.1	7.3±3.8	4.8±4.0	2.5±2.1	7.7±3.6	5.2±4.0
Cortisol	13.5±7.4	17.2±9.3	3.8±11.7	10.8±4.8	11.1±4.7	0.3±6.0
CMJ	17.9±4.4	16.6±4.3	-1.3±1.2	19.2±4.0	16.6±4.7	-2.6±3.1
Power output	13.9±2.5	13.0±2.5	-0.9±0.8	14.8±2.5	13.1±2.9	-1.7±2.1
Alpha-amylase	205.6±73.4	330.8±112.1	125.2±72.4	151.3±105.4	209.7±88.1	58.4±78.2
Stroop performance	30.4±3.2	35.9±2.7	5.5±2.3	31.7±3.1	33.8±2.3	2.1±2.7
Peripheral RPE baseline	-	-	6.5±1.1	-	-	6.4±0.7
Peripheral RPE 1	-	-	7.8±1.5	-	-	7.2±1.6
Peripheral RPE 2	-	-	10.7±2.4	-	-	8.4±2.9
Peripheral RPE 3	-	-	12.9±2.7	-	-	9.7±3.6
Peripheral RPE 4	-	-	14.0±3.0	-	-	10.2±3.7
Peripheral RPE 5	-	-	14.9±3.3	-	-	10.9±4.0
Central RPE baseline	-	-	6.5±1.5		-	6.6±1.2
Central RPE 1	-	-	7.0±1.7	-	-	8.7±2.4
Central RPE 2	-	-	8.7±2.4	-	-	10.6±3.4
Central RPE 3	-	-	9.4±3.0	-	-	13.1±4.0
Central RPE 4	-	-	10.1±3.1	-	-	13.9±4.3
Central RPE 5	-	-	10.7±3.8	-	-	15.4±4.2

**Table 2:** Inferential statistics data.

Factor	Dependent variable	F	p	Effect size	Direction of efects
Mask Use (MU)	Lactate	0.149	0.705	0.010	-
	Cortisol	0.214	0.650	0.014	-
	CMJ	1.598	0.226	0.096	-
	Alpha-amylase	6.850	0.019	0.314	FFP2/N95 > No mask
	Power output	1.504	0.239	0.091	-
	Stroop performance	10.846	0.005	0.420	FFP2/N95 > No mask
	Peripheral RPE baseline	0.529	0.478	0.034	-
	Peripheral RPE 1	2.935	0.107	0.164	-
	Peripheral RPE 2	16.238	0.001	0.520	FFP2/N95 > No mask
	Peripheral RPE 3	19.399	0.001	0.564	FFP2/N95 > No mask
	Peripheral RPE 4	27.157	0.000	0.644	FFP2/N95 > No mask
	Peripheral RPE 5	18.592	0.001	0.553	FFP2/N95 > No mask
	Central RPE baseline	0.594	0.453	0.038	-
	Central RPE 1	9.487	0.008	0.387	FFP2/N95 < No mask
	Central RPE 2	9.891	0.007	0.397	FFP2/N95 < No mask
	Central RPE 3	27.438	0.000	0.647	FFP2/N95 < No mask
	Central RPE 4	20.500	0.000	0.577	FFP2/N95 < No mask
	Central RPE 5	21.796	0.000	0.592	FFP2/N95 < No mask
Condition Presentation Order (CPO)	Lactate	22.091	0.000	0.596	First FFP2/N95 < First No Mask
	Cortisol	0.267	0.613	0.017	-
	CMJ	0.000	0.999	0.000	-
	Alpha-amylase	3.447	0.083	0.187	-
	Power output	0.024	0.879	0.002	-
	Stroop performance	1.062	0.319	0.066	-
	Peripheral RPE baseline	0.049	0.827	0.003	-
	Peripheral RPE 1	0.137	0.717	0.009	-
	Peripheral RPE 2	0.108	0.747	0.007	-
	Peripheral RPE 3	0.354	0.561	0.023	-
	Peripheral RPE 4	0.078	0.784	0.005	-
	Peripheral RPE 5	0.005	0.943	0.000	-
	Central RPE baseline	0.434	0.520	0.028	-
	Central RPE 1	0.121	0.732	0.008	-
	Central RPE 2	0.158	0.697	0.010	-
	Central RPE 3	0.267	0.613	0.017	-
	Central RPE 4	0.166	0.689	0.011	-
	Central RPE 5	0.196	0.664	0.013	-
MU x CPO	Lactate	0.052	0.823	0.003	-
	Cortisol	3.045	0.101	0.169	-
	CMJ	0.807	0.383	0.051	-
	Alpha-amylase	1.002	0.333	0.063	-
	Power output	0.765	0.395	0.049	-
	Stroop performance	2.753	0.118	0.155	-
	Peripheral RPE baseline	1.105	0.310	0.069	-
	Peripheral RPE 1	2.234	0.156	0.130	-
	Peripheral RPE 2	3.030	0.102	0.168	-
	Peripheral RPE 3	0.075	0.788	0.005	-
	Peripheral RPE 4	0.346	0.565	0.023	-
	Peripheral RPE 5	0.105	0.750	0.007	-
	Central RPE baseline	1.239	0.283	0.076	-
	Central RPE 1	0.225	0.642	0.015	-
	Central RPE 2	1.137	0.303	0.070	-
	Central RPE 3	0.560	0.466	0.036	-
	Central RPE 4	1.292	0.273	0.079	-
	Central RPE 5	0.770	0.394	0.049	-

## Discussion

Statistical analysis showed no significant impact of face mask use on motor performance (i.e., CMJ and power output) or metabolic response (i.e., blood lactate concentration). In addition, while cortisol levels remained unaffected by face mask use, alpha-amylase levels were notably higher with the FFP2/N95 mask. Peripheral RPE scores were consistently higher when using the FFP2/N95 mask, though both conditions showed an increasing trend over time. Interestingly, central RPE values were lower using the FFP2/N95 mask in all measurements, except the baseline. This indicates a significant effect of mask use on both central and peripheral RPE.

The findings on selective attention are particularly intriguing when presented concurrently with those of RPE. Selective attention efficiency was significantly better and central fatigue (i.e., mental) was significantly lower in the FFP2/N95 condition (vs. no face mask). This aligns with previous research on mental fatigue [e.g., 46].

The lack of a main effect of condition presentation order or its interaction with face mask use indicates a successful experimental design randomization, enhancing the study’s internal validity in its ecological context. These findings add a fresh perspective to existing research, especially concerning mental responses (i.e., central RPE and selective attention) to face mask use. Given the varied methodologies in prior face mask use studies during physical activity, direct comparisons with this study might not be appropriate. Such comparisons are better suited for future systematic reviews or meta-analyses. For more robust findings, future studies should consider larger sample sizes, extended follow-ups, diverse participants, and varied measurement techniques.

At this point, it’s crucial to note the limitations of this study. The experimental design didn’t consider hormonal factors such as menstrual cycle phase, hormonal contraceptive use, or menopause stage [17]. Hematological [47] and ventilatory [48] responses were also not accounted for. Furthermore, the results are specific to the studied population (i.e., active healthy women), the given training load (i.e., prescribed HIIT), and the face mask type (i.e., FFP2/N95). Therefore, care should be taken when generalizing these findings.

In light of the above, we propose that flow restriction might be a viable tool for boosting selective attention efficiency in simulated competitive scenarios for performance enhancement. While the Stroop task appears to activate a fronto-temporo-parietal brain network involved in decision-making [49], it isn’t sport-specific. Future studies and professionals should explore the potential effects of flow restriction in sport-specific decision-making tasks.

Also, in rehabilitation and athletic performance recovery, flow restriction during HIIT on a stationary bike could simulate high-demand scenarios for those unable to do high-impact exercises like sprinting. While practitioners should tailor training programs to an athlete’s specific sport demands [50], future research could investigate if using flow restriction can be an effective strategy to increase training load during rehabilitation.

All in all, this study offers groundbreaking insights into the mental, physical and psychophysiological responses to FFP2/N95 mask use during a HIIT in active healthy women, enriching existing research in the field. Given the findings presented above, both researchers and practitioners are advised to take into account the potential central and peripheral responses to face mask use during exercise. This nuanced understanding could have far-reaching implications for public health policies and training programs that require or could benefit from face mask usage.

## Supporting information

S1 Data (SAV)
